# Assessing physicians’ and nurses’ experience of dying and death in the ICU: development of the CAESAR-P and the CAESAR-N instruments

**DOI:** 10.1186/s13054-020-03191-z

**Published:** 2020-08-25

**Authors:** Florence Boissier, Valérie Seegers, Amélie Seguin, Stéphane Legriel, Alain Cariou, Samir Jaber, Jean-Yves Lefrant, Thomas Rimmelé, Anne Renault, Isabelle Vinatier, Armelle Mathonnet, Danielle Reuter, Olivier Guisset, Christophe Cracco, Jacques Durand-Gasselin, Béatrice Éon, Marina Thirion, Jean-Philippe Rigaud, Bénédicte Philippon-Jouve, Laurent Argaud, Renaud Chouquer, Laurent Papazian, Céline Dedrie, Hugues Georges, Eddy Lebas, Nathalie Rolin, Pierre-Edouard Bollaert, Lucien Lecuyer, Gérald Viquesnel, Marc Leone, Ludivine Chalumeau-Lemoine, Maité Garrouste-Orgeas, Elie Azoulay, Nancy Kentish-Barnes

**Affiliations:** 1 Medical Intensive Care, University Hospital of Poitiers, Poitiers, France; 2 INSERM CIC 1402 (ALIVE group), Poitiers University, Poitiers, France; 3 Data Management Research Department DRCI, Angers Hospital and SFR ICAT, University of Angers, Angers, France; 4 Medical Intensive Care, Caen University Hospital, Caen, France; 5 Intensive Care, Versailles Hospital, Versailles, France; 6 Medical Intensive Care, Assistance Publique Hôpitaux de Paris, Cochin University Hospital, Paris, France; 7 Paris Descartes University, Paris, France; 8 Saint Eloi Hospital, Centre Hospitalier Universitaire Montpellier, Anesthesia and Critical Care Department B, Montpellier, France; 9 PhyMedExp, University of Montpellier, Montpellier, France; 10 INSERM U1046, CNRS UMR 9214, Montpellier, France; 11 Anesthesia and Intensive Care, Carémeau University Hospital, Nîmes, France; 12 Nîmes University, Nîmes, France; 13 Anaesthesia and Intensive Care Medicine, Hospices Civils de Lyon, Edouard Herriot University Hospital, Lyon, France; 14 University Claude Bernard Lyon 1, Lyon, France; 15 Medical Intensive Care, Cavale Blanche University Hospital, Brest, France; 16 Medical Intensive Care, Les Oudairies Hospital, La Roche Sur Yon, France; 17 Medical Intensive Care, Hospital de la Source, Orléans, France; 18 Medical Intensive Care, Assistance Publique Hôpitaux de Paris, Saint Louis University Hospital, Paris, France; 19 Medical Intensive Care, Saint André University Hospital, Bordeaux, France; 20 Intensive Care, Angoulême Hospital, Angoulême, France; 21 Anaesthesia and Intensive Care, Sainte Musse Hospital, Toulon, France; 22 Anaesthesia and Intensive Care, La Timone University Hospital, Marseille, France; 23 Medical Intensive Care, Victor Dupouy Hospital, Argenteuil, France; 24 Medical Intensive Care, Dieppe Hospital, Dieppe, France; 25 Intensive Care, Roanne Hospital, Roanne, France; 26 Medical Intensive Care, Hospices Civils de Lyon, Edouard Herriot University Hospital, Lyon, France; 27 Lyon Est University, Lyon, France; 28 Intensive Care, Annecy Hospital, Annecy, France; 29 Medical Intensive Care, Assistance Publique Hôpitaux de Marseille, Hôpital Nord, Marseille, France; 30 Aix-Marseille University, Marseille, France; 31 Intensive Care, Roubaix Hospital, Roubaix, France; 32 Intensive Care, Chatilliez Hospital, Tourcoing, France; 33 Intensive Care, Bretagne Atlantique Hospital, Vannes, France; 34 Medical Intensive Care, Groupe Hospitalier Sud Ile de France, Melun, France; 35 Medical Intensive Care, Nancy University Hospital, Nancy, France; 36 Lorraine University, Nancy, France; 37 Medical Intensive Care, Sud Francilien Hospital, Evry, France; 38 Surgical Intensive Care, Caen University Hospital, Caen, France; 39 Anaesthesia and Intensive Care, Assistance Publique Hôpitaux de Marseille, Hôpital Nord, Marseille, France; 40 Intensive Care, Gustave Roussy Institut, Villejuif, France; 41 Intensive Care, Saint Joseph Hospital, Paris, France; 42 Biostatistics and Clinical Epidemiology Research Team, U1153, INSERM, Paris Diderot Sorbonne University, Paris, France; 43 Famiréa Research Group, Assistance Publique Hôpitaux de Paris, Saint Louis University Hospital, Paris, France; 44 Medical ICU, Hôpital Saint-Louis, 1 avenue Claude Vellefaux, 75010 Paris, France

**Keywords:** Caregivers, Intensive care unit, End-of-life experience, Death

## Abstract

**Background:**

As an increasing number of deaths occur in the intensive care unit (ICU), studies have sought to describe, understand, and improve end-of-life care in this setting. Most of these studies are centered on the patient’s and/or the relatives’ experience. Our study aimed to develop an instrument designed to assess the experience of physicians and nurses of patients who died in the ICU, using a mixed methodology and validated in a prospective multicenter study.

**Methods:**

Physicians and nurses of patients who died in 41 ICUs completed the job strain and the CAESAR questionnaire within 24 h after the death. The psychometric validation was conducted using two datasets: a learning and a reliability cohort.

**Results:**

Among the 475 patients included in the main cohort, 398 nurse and 417 physician scores were analyzed. The global score was high for both nurses [62/75 (59; 66)] and physicians [64/75 (61; 68)]. Factors associated with higher CAESAR-Nurse scores were absence of conflict with physicians, pain control handled with physicians, death disclosed to the family at the bedside, and invasive care not performed. As assessed by the job strain instrument, low decision control was associated with lower CAESAR score (61 (58; 65) versus 63 (60; 67), *p* = 0.002).

Factors associated with higher CAESAR-Physician scores were room dedicated to family information, information delivered together by nurse and physician, families systematically informed of the EOL decision, involvement of the nurse during implementation of the EOL decision, and open visitation. They were also higher when a decision to withdraw or withhold treatment was made, no cardiopulmonary resuscitation was performed, and the death was disclosed to the family at the bedside.

**Conclusion:**

We described and validated a new instrument for assessing the experience of physicians and nurses involved in EOL in the ICU. This study shows important areas for improving practices.

## Background

As an increasing number of deaths occur in the intensive care unit (ICU), studies have sought to describe, understand, and improve end-of-life care in this setting [1–5]. Most of these studies are centered on the patient’s and/or the relatives’ experience and have permitted to highlight elements of care that need improvement. Fewer studies have evaluated the healthcare professionals’ experience and perception of dying and death. Whether in the hospital in general [6] or in the ICU in particular [4, 7–10], these studies show a discrepancy between physicians’ and nurses’ experience and perception of end-of-life care—the latter generally expressing less satisfaction concerning pain control and quality of discussions and decision-making.

Among end-of-life research projects, tools have been developed to evaluate the quality of dying and death in the ICU, such as the Quality Of Dying and Death (QODD) instrument [11]. Quality of dying and death is defined as “the degree to which a person’s preferences for dying and the moment of death agree with observations of how the person actually died as reported by others” [12]. The quality of dying and death is subjectively determined with multiple factors that influence its evaluation, including culture, type and stage of disease, social and, importantly, professional role in the dying experience. A study comparing QODD ratings by relatives and by healthcare professionals [9] shows that relatives and attending physicians give the most favorable ratings of death, while nurses and residents provide less favorable ratings. Significant differences between these groups are notable on items related to patient autonomy. However, this tool was designed and validated in the USA, where hospital and end-of-life culture, and physicians’, nurses’, relatives’, and patients’ roles are different than in Europe [4, 13].

Thus, the French CAESAR project aimed to develop an instrument to assess the experience of relatives of patients who died in the ICU as well as the experience of healthcare professionals, physicians, and nurses. The relatives’ instrument was validated in a previous publication and presents valuable psychometric properties [5]. This 15-item questionnaire covered the patient’s preferences and values, interactions with/around the patient, and family satisfaction. Relatives’ lower scores were associated with greater risks of anxiety and depression at 3 months; post-traumatic stress-related symptoms at 3, 6, and 12 months; and complicated grief at 6 and 12 months.

Here, our objective was to develop and validate two tools specifically designed to assess the overall experience of physicians and nurses caring for patients who died in the ICU. We used a mixed methodology to create the instrument that was then validated in a multicenter prospective study in 41 French ICUs.

## Participants and methods

The study was approved by the institutional review board of the Paris-North Hospitals (IRB00006477, approval#11019), Paris 7 University. Informed consent was obtained from each relative before study inclusion.

Physicians and nurses were invited to participate and to complete the CAESAR tool on a voluntary basis. They received information about the study, and those who did not wish to participate could decline. No written consent was required.

Before inclusion of patients, in each participating center, the local investigator, together with the head nurse, completed a description of the ICU.

### Development and validation of the CAESAR-P and CAESAR-N instruments

Development of the CAESAR instrument was described in a previous publication [5]. A 33-item questionnaire for physicians and nurses was validated in a multicenter prospective study conducted from July 2011 to July 2013 in 41 French ICUs belonging to the FAMIREA network. The 33 items fell into three domains: the patient (preparation for death, whole person concerns, symptoms, personal care, and treatment preferences), interactions with and around the patient (quality of communication between ICU team and the patient and between the ICU team and the relatives, particularly whether conflicts arose), and family needs and satisfaction.

In each ICU, the intensivist included consecutive adults who died after at least 48 h in the ICU and for whom the relatives had visited at least once. For each patient, the physician and the nurse in charge of the patient were asked to complete the instrument within 24 h after the death. At the end of the instrument, participants were also asked to complete information such as age, sex, years of experience in the ICU, and religious beliefs. As described elsewhere, relatives completed the tool 21 days after the patient’s death [5].

After full data completion, 7 investigators (4 physicians, 1 psychologist, 1 sociologist, and 1 biostatistician) for the physicians’ instrument (CAESAR-P), and 6 investigators (2 nurses, 1 physician, 1 psychologist, 1 sociologist and 1 biostatistician) for the nurses’ instrument (CAESAR-N), worked to reduce the instrument from 33 to 15 items, based on clinical relevance and item distribution, discrimination, and redundancy [5]. The remaining 15 items (Table 1) were analyzed to explore the psychometric characteristics of the instrument, among physicians and among nurses separately. The psychometric validation was conducted using two sets of instruments: those completed by healthcare professionals for patients included in the CAESAR study [5] as the learning cohort (derivation set), and those completed by healthcare professionals for patients who had the same inclusion criteria, but who were not included in the CAESAR study, as the reliability cohort (validation set). These data were recorded at the beginning of the study period before inclusion started in the CAESAR study. Briefly, factorial validity was assessed in the derivation set and validation set separately by determining the dimensional structure of the final 15-item CAESAR questionnaire. To this end, we used maximum likelihood factor analysis with varimax rotation. The number of factors was determined from the observation of the scree plots and value of simulations [14–16]. Internal consistency was deemed acceptable when Cronbach’s alpha [17] was in the 0.70–0.95 range [18]. For the final 15-item tool, the item scores (range 1–5) were summed to obtain a global score (15–75). Each item included a written description and a score on a 5-point scale: 1, traumatic; 2, painful; 3, difficult; 4, acceptable; and 5, comforting. As a result, if a clinician rates all questions at 1, his/her total score will be 15, reflecting an overall traumatic experience. If a clinician rates all questions at 5, his/her total score will be 75, reflecting a very positive experience. Based on these calculations, scores between 60 and 75 reflect an overall positive experience; scores lower than 45 reflect an overall negative experience; scores between 45 and 60 are considered intermediary, reflecting that some elements of the end-of-life process were experienced negatively.

**Table 1:** Physician and nurse CAESAR scores

**Items for physicians and nurses**	**Physician**, median score (IQR)	**Nurse**, median score (IQR)
**1. Was an EOL palliative care approach clearly decided for the patient?** Please rate this experience: 1, traumatic; 2, painful; 3, difficult; 4, acceptable; 5, comforting	4 (4–5)	4 (4–5)
**2. Was the decision to withhold or withdraw treatment clearly documented in the medical report?** Please rate this experience: 1, traumatic; 2, painful; 3, difficult; 4, acceptable; 5, comforting	4 (4–5)	4 (4–5)
**3. Do you think the patient received excessive or futile care?** Please rate this experience: 1, traumatic; 2, painful; 3, difficult; 4, acceptable; 5, comforting	5 (4–5)	4 (4–5)
**4. Was the patient able to communicate with you during his/her ICU stay?** Please rate this experience: 1, traumatic; 2, painful; 3, difficult; 4, acceptable; 5, comforting	4 (4–4)	4 (4–4)
**5. Was the patient’s pain under control?** Please rate this experience: 1, traumatic; 2, painful; 3, difficult; 4, acceptable; 5, comforting	4 (4–5)	5 (4–5)
**6. Was the patient able to breathe comfortably?** Please rate this experience: 1, traumatic; 2, painful; 3, difficult; 4, acceptable; 5, comforting	4 (4–4)	4 (3–4)
**7. In your opinion, was the patient’s dignity respected?** Please rate this experience: 1, traumatic; 2, painful; 3, difficult; 4, acceptable; 5, comforting	5 (4–5)	5 (4–5)
**8. Did the relatives pay regular visits to the patient?** Please rate this experience: 1, traumatic; 2, painful; 3, difficult; 4, acceptable; 5, comforting	5 (4–5)	5 (4–5)
**9. Did the ICU team discuss the patient’s EOL wishes with the patient him/herself or with the relatives?** Please rate this experience: 1, traumatic; 2, painful; 3, difficult; 4, acceptable; 5, comforting	4 (4–5)	4 (3–5)
**10. Were the relatives at the patient’s bedside at the time of death?** Please rate this experience: 1, traumatic; 2, painful; 3, difficult; 4, acceptable; 5, comforting	4 (4–5)	4 (3–4)
**11. During the patient’s ICU stay, did the relatives receive support from a psychologist?** Please rate this experience: 1, traumatic; 2, painful; 3, difficult; 4, acceptable, 5, comforting	4 (4–4)	4 (4–5)
**12. Are you satisfied with the patient’s overall quality of dying and death?** Please rate this experience: 1, traumatic; 2, painful; 3, difficult; 4, acceptable; 5, comforting	4 (4–5)	4 (4–5)
**13. If the patient had been your relative, would you have been satisfied with his/her EOL?** Please rate this experience: 1, traumatic; 2, painful; 3, difficult; 4, acceptable; 5, comforting	4 (4–5)	4 (3–5)
**Specific physician items**		
**14. Were the relatives able to say goodbye to the patient?** Please rate this experience: 1, traumatic; 2, painful; 3, difficult; 4, acceptable; 5, comforting	5 (4–5)	
**15. Did you experience conflict with the patient and/or the relatives?** Please rate this experience: 1, traumatic; 2, painful; 3, difficult; 4, acceptable; 5, comforting	5 (4–5)	
**Specific nurse items**		
**14. Were the relatives able to have physical contact (touch, hug) with the patient?** Please rate this experience: 1, traumatic; 2, painful; 3, difficult; 4, acceptable, 5, comforting	4 (4–5)	
**15. Were you present at the patient’s bedside at the time of death?** Please rate this experience: 1, traumatic; 2, painful; 3, difficult; 4, acceptable, 5, comforting	4 (3–4)	

*EOL* end-of-life

We also collected ICU, healthcare professionals’, patients’, and end-of-life characteristics.

### Job strain evaluation

Physicians’ and nurses’ job strain was evaluated using an instrument that explores three domains: job demand (3 questions), control (5 questions), and social support (4 questions) [19–21]. High job demand, low decision control, and poor social support were graded as high for a score at 2–3, at 3–5, and at 3–4, respectively. Global job strain score was considered high (i.e., job strain positive) if there were 2 or 3 positive items among high job demand, poor decision control, and poor social support.

### Statistical analysis

Statistical analyses were performed using the R 3.1 package with the “psy” package [22].

Quantitative data were described as median (25e–75e percentiles, i.e., interquartile range); comparisons between groups were tested using ANOVA test. Binary and categorical data were described as number and percentages. No imputation for missing data was performed. To assess the number of dimension identified in the survey, a principal component analysis and the corresponding scree plot were used. The cutoff was determined using a set of simulations that illustrated the amount of variance that may be expected by chance alone [14]. The number of factors over the simulations may be of interest. The internal consistency was assessed with the Cronbach alpha (95% bootstrap confidence interval), split-half, and composite reliability. The association between job strain results and CAESAR-P and CAESAR-N scores was assessed using ANOVA test, according to the 3 dimensions. The center effect on CAESAR-N and CAESAR-P scores was assessed using a mixed regression model. All statistical tests were two-sided, and *p* values of 0.05 or less were considered significant.

## Results

### Study population

Patients’, ICUs’, and management of end-of-life characteristics are summarized in Supplemental Table 1. Physician and nurses’ characteristics are summarized in Supplemental Table 2. Among the 4607 patients admitted to the 41 participating ICUs during the study period, 875 (19%) died, including 228 who met exclusion criteria, 104 for whom the opportunity for inclusion was missed, and 68 whose relatives refused participation [5]. For each of the remaining 475 (54%) patients, one relative was included. Within 24 h of the death, the physician and the nurse in charge of the patient completed the 33-item instrument as well as the job strain (completed only once during the study period).

### CAESAR scores

Among the 475 patients included, 398 nurse scores and 417 physician scores were analyzed.

The response rates were 441/475 (92.8%) for nurses and 446/475 (93.9%) for physicians. Among these, respectively 398/441 (90.2%) and 417/446 (93.5%) were fully completed, allowing score calculation and analysis.

The median global CAESAR score was 62/75 (59; 66) for nurses and 64/75 (61; 68) for physicians; the proportion of surveys with scores higher than 60/75 were 248/398 (62.3%) for nurses and 313/417 (75.1%) for physicians. There was no center effect for nurses (*p* 0.28), but a center effect was detected for physicians’ scores (*p* < 0.001).

**Fig. 1:**
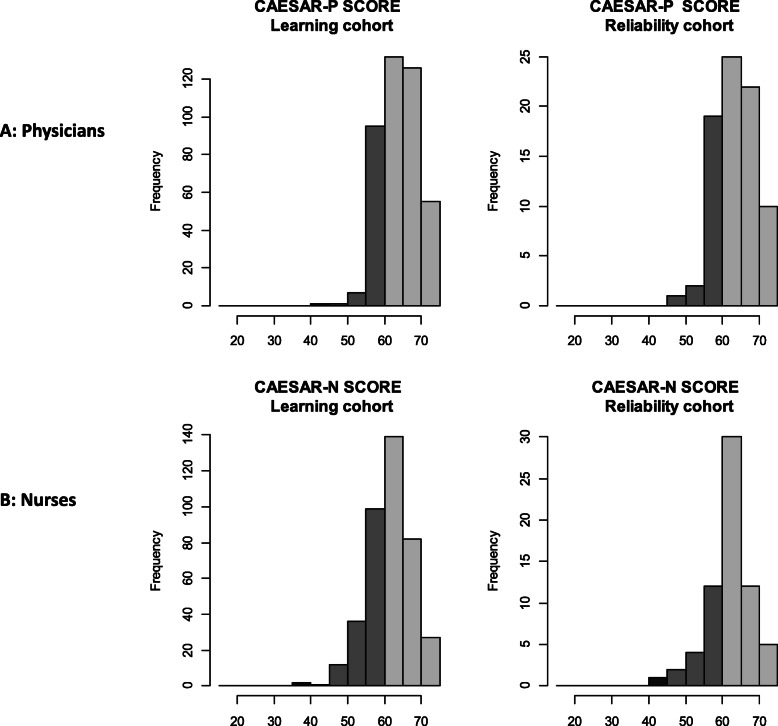
Distribution of the global CAESAR-P and CAESAR-N scores. Histograms: dark gray, score < 45; gray, score 45–60; light gray, score > 60

**Fig. 2:**
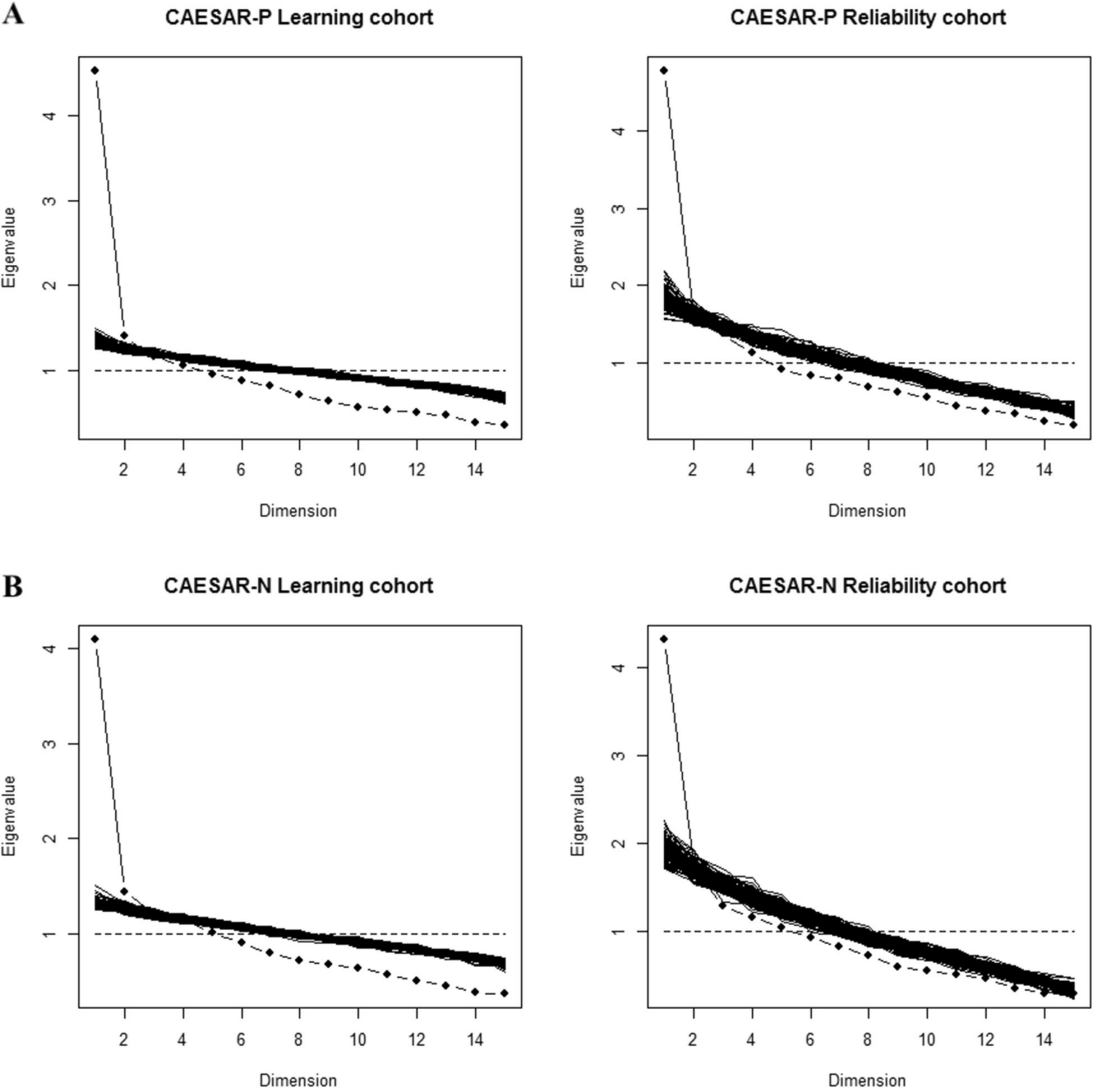
Psychometric validation of the CAESAR-P and CAESAR-N. The factorial analyses and their corresponding scree plots are presented in **a** and **b**. In both cohorts and in both questionnaires, the first factor on its own explained a substantial amount of the item variance and no other factor appears of comparable importance, compared to the value of simulations. The scale is thus fairly homogeneous, if not strictly unidimensional

Supplemental Tables 3 and 4 depict the distribution of individual item scores for physicians and nurses, respectively. Figure 1 a and b depict the distribution of global scores for physicians and nurses, respectively. The factorial analysis of the main sample, assessed by scree plots, was consistent with a single dimension for both questionnaires, in the learning cohort and in the reliability cohort (Fig. 2a, b). Internal consistency was acceptable for both scales (Supplemental Tables 5 and 6).

### Factors associated with the CAESAR-P and the CAESAR-N scores

Physicians’ CAESAR-P score (Table 2) was higher when there was a room dedicated to family information, when the information was delivered together by nurse and physician, when families were systematically informed of the EOL decision rather than occasionally, when implementation of the EOL decision involved the nurse and physician rather than the physician only, and when the ICU was open 24 h/24 h. The physicians’ score was also higher when the patient had a McCabe score [23] at 1 rather than higher (i.e., no fatal expectancy); was not immunocompromised; had no dementia, liver failure, nor hematological disease; and did not require psychotropic medication. The score was higher when a decision to withdraw or withhold treatment was made, when no cardiopulmonary resuscitation was performed, and when the death was disclosed to the family at the bedside rather than by phone or upon arrival at the ICU.

**Table 2:** Factors associated with the nurses’ and physicians’ CAESAR scores

	Score, median (IQR)	*p* value
**Nurse scores**		
**Conflict with physicians**		0.02
Yes	61 (58–65)	
No	63 (59–67)	
**Pain management**		0.02
By nurse	62 (59–66)	
By physician	63 (60–68)	
**Death disclosure to family**		0.006
By phone or upon arrival at the ICU	61 (58–65)	
At the bedside	63 (59–67)	
**Surgery**		0.03
Yes	60 (57–66)	
No	62 (59–66)	
**Chest tube**		0.02
Yes	61 (57–64)	
No	62 (59–67)	
**Bronchoscopy**		0.02
Yes	62 (58–65)	
No	62 (59–67)	
**Job strain: low decision control**		0.002
Yes	61 (58–65)	
No	63 (60–67)	
**Physician scores**		
**McCabe**		< 0.001
1	66 (62–70)	
≥ 2	63 (60–67)	
**Immunocompromised**		0.003
Yes	63 (60–67)	
No	65 (61–69)	
**Dementia**		0.02
Yes	59 (59–62)	
No	64 (61–68)	
**Liver failure**		0.04
Yes	63 (60–66)	
No	64 (61–68)	
**Hematological disease**		0.005
Yes	62 (58–64)	
No	65 (61–68)	
**Psychotropic medication**		0.03
Yes	63 (60–67)	
No	65 (61–69)	
**Decision to withdraw or withhold treatment**		< 0.001
Withdraw	66 (61–69)	
Withhold	63 (60–67)	
Neither	62 (60–66)	
**Cardiopulmonary resuscitation**		< 0.001
Yes	60 (58–63)	
No	65 (61–68)	
**Death disclosure to family**		0.002
By phone or upon arrival	64 (60–67)	
At bedside	65 (61–69)	
**Room dedicated to family information**		0.005
Yes	65 (61–69)	
No	62 (60–66)	
**Family information delivered together by nurse and physician**		0.002
Yes	65 (61–69)	
No	62 (60–66)	
**Systematic family information of an EOL decision**		0.01
Yes	65 (61–69)	
No	62 (60–66)	
**Implementation of decision**		0.03
By physician only	63 (60–67)	
By physician and nurse	65 (61–69)	
**24-h visiting in the ICU**		0.007
Yes	65 (61–70)	
No	64 (60–68)	

The distribution of CAESAR scores according to the following characteristics was summarized using median and interquartile range. Comparison of scores between characteristic modalities was performed using ANOVA test; a *p* value < 0.05 was considered statistically significant

The nurse CAESAR-N score (Table 2) was significantly higher in the absence of conflict with physicians, when pain control was handled by physicians rather than by nurses alone, when the death was disclosed to the family at the bedside rather than by phone or upon arrival at the ICU, and when invasive care such as surgery, chest tube, or bronchoscopy was not performed.

### Job strain

Job strain was evaluated among 231 physicians and 379 nurses. Ten percent of physicians had a positive job strain. Among the 3 domains (job demand, decision control, and social support), the most frequent complaint for physicians was a high job demand (38.2%), but there was no association between the CAESAR score and job strain for physicians. Twenty-seven percent of nurses had a positive job strain. The most frequent complaint for nurses was a low decision control (54.2%) and a high job demand (44.5%). Low decision control was associated with lower CAESAR score for nurses (61 (58; 65) versus 63 (60; 67), *p* = 0.002).

## Discussion

This multicenter study allowed us to develop and validate two 15-item CAESAR questionnaires (CAESAR-P and CAESAR-N) designed to measure self-reported experience of caregivers about patients’ end-of-life in the ICU, as we did previously for relatives [5].

Both tools (for nurses and for physicians) showed good internal consistency and a single dimension. The global score was high for both nurses [62/75 (59; 66)] and physicians [64/75 (61; 68)], reflecting global satisfaction regarding dying and death in the ICU, in keeping with previous studies [4, 24]. The nurses’ and the physicians’ results cannot however be strictly compared, as 2 questions differ between the two instruments.

Previous studies aiming at evaluating quality of dying and death [4, 9, 25] used the QODD, which was designed and validated in the USA, where hospital and end-of-life culture, and physicians’, nurses’, relatives’, and patients’ roles are different than in Europe. However, this tool has recently been used in Europe: in a study by Gerritsen et al. [25], the single-item QODD summary score was significantly higher for nurses in the Netherlands than in the USA, probably due to organizational (presence of a physician in the ICU and more often at the bedside in the Netherlands) and cultural differences.

In France, nurses’ perception of dying and death was evaluated over 10 years ago in hospitals [6] and in ICUs [7], showing poor ratings of quality of death and of end-of-life decisions. However, French legislation changed in 2005 and 2016, allowing withdrawing and withholding treatment, as well as palliative sedation, which has positively modified end-of-life culture in ICUs: our results are consistent with these improvements. In a more recent multicenter French study [24], nurses rated the end-of-life of their patients under mechanical ventilation at 8 on a scale from 1 (worst) to 10 (best), concordant with our findings. Research thus shows high rating of dying in the ICU, whether in Europe or the USA.

In our current study, nurses’ experience of the patient’s end-of-life in the ICU was worse in case of conflict with physicians. In a previous European study about ICU conflicts [20], end-of-life care was one of the main reported sources of conflict. In these situations, the principal sources of conflict were lack of psychological support, absence of unit-level meetings, and problems with the decision-making process. Two factors were associated with less conflict, i.e., symptom control performed jointly by physicians and nurses, and routine unit-level meetings. In our study, these two factors were also associated with a better experience of end-of-life for nurses (pain control handled by nurses rather than by physicians only, relatives’ information delivered together by nurse and physician) and for physicians (implementation of the end-of-life decision involving the nurse rather than the physician alone). Indeed high-quality end-of-life care requires good inter-professional collaboration and communication [26]. The job strain evaluation shows that for nurses, low decision control was associated with lower CAESAR score, highlighting the importance of valuing nurses’ involvement in decision-making processes. Physicians’ job strain was not associated with CAESAR score.

For both nurses and physicians, a better experience of end-of-life was associated with an absence of invasive care (considered aggressive in this setting): for nurses, absence of invasive care such as surgery, chest tube, or bronchoscopy; for physicians, decision to withhold or withdraw treatment, and absence of cardiopulmonary resuscitation—factors concordant with other study results [8, 9]. Absence of overaggressive treatment at the end-of-life can also be interpreted as a sign of good inter-professional communication as well as good communication with patients and family members [26, 27]. Early integration of palliative care that focuses on reducing suffering among patients with serious illness and their family members is recommended: in a study from the USA, nurse-assessed quality of dying was significantly improved with an intervention to integrate palliative care in the ICU [28].

Finally, good communication with family members and their presence in the ICU were associated with a better experience of patients’ end-of-life for nurses and physicians (communication at the bedside rather by phone, presence of a room dedicated to family information, systematic information of decision rather than occasional, open visiting hours) and are key elements in end-of-life care, as shown in previous studies [8, 25, 29]. Families are no longer simple visitors in the ICU: they play important roles and should now be considered by the ICU team as active partners, including in end-of-life situations [30].

This study has several limitations. First, all participating ICUs were in France and the findings may not be pertinent in different cultural settings. However, the large number of participating ICUs and clinicians, the very high response rate, and the validation of the results in a reliability cohort support the robustness of the data. Second, authors are aware of the potential issues with using an older dataset, specifically the potential changes of EOL practices across the world. However, use of the tool will permit to compare and describe changes across time and countries. Third, the global score was high in both groups of clinicians and the score differences were low and may not be meaningful to clinicians. However, this is true for other end-of-life tools used in Europe, such as the QODD [25]. Lastly, the description of the score on a 5-point scale (1, traumatic; 2, painful; 3, difficult; 4, acceptable; 5, comforting) raises a challenge in terms of measuring subjective responses. While pilot testing did not yield any difficulties between scoring on the scale, this is something to consider in future translations of this tool.

## Conclusion

In summary, we described and validated two new instruments for assessing nurses’ and physicians’ experience of end-of-life in the ICU. Our study shows factors associated with a better experience that include quality communication, both with family members and inter-professional communication and collaboration; family presence in the ICU; and avoidance of aggressive care. These results will help design future interventional studies aimed at improving end-of-life care in the ICU.

## Supplementary information

**Additional file 1: Supplemental Table 1.** Characteristics of ICU, end-of-life management and patients.

**Additional file 2: Supplemental Table 2.** Characteristics of physicians and nurses.

**Additional file 3: Supplemental Table 3.** Psychometric validation of the physician questionnaire: distribution of individual item scores.

**Additional file 4: Supplemental Table 4.** Psychometric validation of the nurse questionnaire: distribution of individual item scores.

**Additional file 5: Supplemental Table 5.** Psychometric validation of the physician questionnaire: Measurement error: internal consistency.

**Additional file 6: Supplemental Table 6.** Psychometric validation of the nurse questionnaire: Measurement error: internal consistency.

## Data Availability

Not applicable
