# Supplementary material for: Assessing physicians’ and nurses’ experience of dying and death in the ICU: development of the CAESAR-P and the CAESAR-N instruments
Source: Crit Care. 2020 Aug 25;24:521. doi: 10.1186/s13054-020-03191-z (PMC7448438; doi:10.1186/s13054-020-03191-z)
Supplement: Supplementary file 1 — Additional file 1: Supplemental Table 1. Characteristics of ICU, end-of-life management and patients. [file 13054_2020_3191_MOESM1_ESM.docx]

**Supplemental Table 1. Characteristics of ICU, end-of-life management and patients**

| **ICU characteristics** | **N=41** |
| --- | --- |
| General ICU | 24 (58.5%) |
| Psychologist available | 15 (36.5%) |
| 24-hour visiting | 14 (34.1%) |
| **ICU end-of-life management** | **N=41** |
| Systematic/frequent multidisciplinary meetings to discuss EOL decisions | 33 (80.4%) |
| End-of-life pain control managed by nurses and physicians together | 33 (80.4%) |
| Relatives systematically informed of the decision to withhold or withdraw treatment | 38 (92.6%) |
| Decision to withhold or withdraw treatment implemented by  physician and nurse together | 25 (60.9%) |
| Post-death meetings with the family organized in the ICU | 6 (14.6%) |
| **Patients** | **N= 475** |
| IGS2, median (IQR) | 58 (44-71) |
| Medical admission | 356 (75%) |
| Length of ICU stay (days), median (IQR) | 7 (4-16) |
| Mechanical ventilation | 337 (71%) |
