# Supplementary material for: Assessing physicians’ and nurses’ experience of dying and death in the ICU: development of the CAESAR-P and the CAESAR-N instruments
Source: Crit Care. 2020 Aug 25;24:521. doi: 10.1186/s13054-020-03191-z (PMC7448438; doi:10.1186/s13054-020-03191-z)
Supplement: Supplementary file 2 — Additional file 2: Supplemental Table 2. Characteristics of physicians and nurses. [file 13054_2020_3191_MOESM2_ESM.docx]

**Supplemental Table 2**: Characteristics of physicians and nurses

|  | **Physicians** | **Nurses** | p.value |
| --- | --- | --- | --- |
| n | 203* | 350* |  |
| **Years of experience in this ICU (%)** |  |  | <0.001 |
| Less than 2 years | 89/200 (44.5) | 130/338 (38.5) |  |
| 2 - 5 years | 43/200 (21.5) | 116/338 (34.3) |  |
| 5 - 10 years | 25/200 (12.5) | 55/338 (16.3) |  |
| More than 10 years | 43/200 (21.5) | 37/338 (10.9) |  |
| **Years of ICU experience (%)** |  |  | <0.001 |
| Less than 2 years | 38/199 (19.1) | 106/339 (31.3) |  |
| 2 - 5 years | 55/199 (27.6) | 117/339 (34.5) |  |
| 5 - 10 years | 46/199 (23.1) | 71/339 (20.9) |  |
| More than 10 years | 60/199 (30.2) | 45/339 (13.3) |  |
| **Gender, male (%)** | 135 (67.2) | 66/339 (19.5) | <0.001 |
| **Age (mean (SD))** | 37.53 (8.67) | 31.34 (7.65) | <0.001 |
| **Ethnicity (%)** |  |  | 0.067 |
| Mainland France | 181/197 (91.4) | 319/338 (94.4) |  |
| Northern Africa | 4/197 (2.0) | 4/338 (1.2) |  |
| Africa | 1/197 (0.5) | 3/338 (0.9) |  |
| Europe | 9/197 (4.5) | 5/338 (1.5) |  |
| Asia | 2/197 (1.0) | 1/338 (0.3) |  |
| DOM-TOM | 0 /197 (0.0) | 6/338 (1.8) |  |
| Other | 1/197 (0.5) | 0/338 (0.0) |  |
| **Religious Beliefs (%)** | 71/197 (36.0) | 143/339 (42.2) | 0.191 |
| **Children (%)** | 119/200 (59.5) | 145/338 (42.9) | <0.001 |
| **Lost a loved-one in the ICU (%)** | 50/200 (25.0) | 61/328 (18.0) | 0.067 |

p-value were computed using exact Fisher test for Geographic origin and χ² test for other characteristics

*417 physician-CAESAR instruments were completed by 203 physicians; 398 nurse-CAESAR instruments were completed by 350 nurses
