# Supplementary material for: Assessing physicians’ and nurses’ experience of dying and death in the ICU: development of the CAESAR-P and the CAESAR-N instruments
Source: Crit Care. 2020 Aug 25;24:521. doi: 10.1186/s13054-020-03191-z (PMC7448438; doi:10.1186/s13054-020-03191-z)
Supplement: Supplementary file 3 — Additional file 3: Supplemental Table 3. Psychometric validation of the physician questionnaire: distribution of individual item scores. [file 13054_2020_3191_MOESM3_ESM.docx]

**Supplemental Table 3:**

**Psychometric validation of the physician questionnaire: distribution of individual item scores**

|  | **Learning Cohort (446 instruments)** | | | | | |  |  | **Reliability Cohort (90 instruments)** | | | | | |
| --- | --- | --- | --- | --- | --- | --- | --- | --- | --- | --- | --- | --- | --- | --- |
| Question | 1 | 2 | 3 | 4 | 5 | <NA> |  | Question | 1 | 2 | 3 | 4 | 5 | <NA> |
| 1 | 0 (0%) | 4 (0,9%) | 31 (7%) | 249 (55,8%) | 159 (35,7%) | 3 (0,7%) |  | 1 | 0 (0%) | 0 (0%) | 3 (3,4%) | 60 (68,2%) | 25 (28,4%) | 2 (2,2%) |
| 2 | 0 (0%) | 0 (0%) | 13 (2,9%) | 265 (59,4%) | 161 (36,1%) | 7 (1,6%) |  | 2 | 0 (0%) | 0 (0%) | 1 (1,2%) | 54 (62,8%) | 31 (36%) | 4 (4,4%) |
| 3 | 0 (0%) | 1 (0,2%) | 5 (1,1%) | 212 (47,5%) | 223 (50%) | 5 (1,1%) |  | 3 | 0 (0%) | 0 (0%) | 1 (1,2%) | 51 (59,3%) | 34 (39,5%) | 4 (4,4%) |
| 4 | 0 (0%) | 1 (0,2%) | 56 (12,6%) | 305 (68,4%) | 81 (18,2%) | 3 (0,7%) |  | 4 | 0 (0%) | 1 (1,1%) | 14 (15,9%) | 62 (70,5%) | 11 (12,5%) | 2 (2,2%) |
| 5 | 0 (0%) | 1 (0,2%) | 32 (7,2%) | 190 (42,6%) | 221 (49,6%) | 2 (0,4%) |  | 5 | 0 (0%) | 1 (1,1%) | 3 (3,4%) | 41 (46,6%) | 43 (48,9%) | 2 (2,2%) |
| 6 | 1 (0,2%) | 5 (1,1%) | 81 (18,2%) | 253 (56,7%) | 101 (22,6%) | 5 (1,1%) |  | 6 | 0 (0%) | 2 (2,3%) | 9 (10,3%) | 51 (58,6%) | 25 (28,7%) | 3 (3,3%) |
| 7 | 0 (0%) | 0 (0%) | 13 (2,9%) | 194 (43,5%) | 237 (53,1%) | 2 (0,4%) |  | 7 | 0 (0%) | 0 (0%) | 3 (3,4%) | 33 (37,5%) | 52 (59,1%) | 2 (2,2%) |
| 8 | 0 (0%) | 1 (0,2%) | 13 (2,9%) | 176 (39,5%) | 251 (56,3%) | 5 (1,1%) |  | 8 | 0 (0%) | 1 (1,1%) | 8 (9,1%) | 33 (37,5%) | 46 (52,3%) | 2 (2,2%) |
| 9 | 0 (0%) | 14 (3,1%) | 49 (11%) | 153 (34,3%) | 226 (50,7%) | 4 (0,9%) |  | 9 | 0 (0%) | 1 (1,1%) | 11 (12,5%) | 36 (40,9%) | 40 (45,5%) | 2 (2,2%) |
| 10 | 0 (0%) | 2 (0,4%) | 23 (5,2%) | 277 (62,1%) | 139 (31,2%) | 5 (1,1%) |  | 10 | 0 (0%) | 0 (0%) | 4 (4,5%) | 55 (62,5%) | 29 (33%) | 2 (2,2%) |
| 11 | 0 (0%) | 6 (1,3%) | 36 (8,1%) | 204 (45,7%) | 194 (43,5%) | 6 (1,3%) |  | 11 | 0 (0%) | 0 (0%) | 9 (10,2%) | 53 (60,2%) | 26 (29,5%) | 2 (2,2%) |
| 12 | 0 (0%) | 21 (4,7%) | 82 (18,4%) | 288 (64,6%) | 50 (11,2%) | 5 (1,1%) |  | 12 | 0 (0%) | 2 (2,3%) | 7 (8%) | 65 (73,9%) | 14 (15,9%) | 6 (6,7%) |
| 13 | 0 (0%) | 3 (0,7%) | 17 (3,8%) | 261 (58,5%) | 160 (35,9%) | 5 (1,1%) |  | 13 | 1 (1,2%) | 0 (0%) | 2 (2,4%) | 56 (66,7%) | 25 (29,8%) | 7 (7,8%) |
| 14 | 0 (0%) | 1 (0,2%) | 3 (0,7%) | 167 (37,4%) | 271 (60,8%) | 4 (0,9%) |  | 14 | 0 (0%) | 2 (2,4%) | 1 (1,2%) | 32 (38,6%) | 48 (57,8%) | 5 (5,6%) |
| 15 | 2 (0,4%) | 24 (5,4%) | 41 (9,2%) | 193 (43,3%) | 176 (39,5%) | 10 (2,2%) |  | 15 | 1 (1,2%) | 5 (5,9%) | 9 (10,6%) | 42 (49,4%) | 28 (32,9%) | 11 (12,2%) |
