# Supplementary material for: Assessing physicians’ and nurses’ experience of dying and death in the ICU: development of the CAESAR-P and the CAESAR-N instruments
Source: Crit Care. 2020 Aug 25;24:521. doi: 10.1186/s13054-020-03191-z (PMC7448438; doi:10.1186/s13054-020-03191-z)
Supplement: Supplementary file 4 — Additional file 4: Supplemental Table 4. Psychometric validation of the nurse questionnaire: distribution of individual item scores. [file 13054_2020_3191_MOESM4_ESM.docx]

**Supplemental table 4:**

**Psychometric validation of the nurse questionnaire:** **distribution of individual item scores**

|  | **Learning cohort (441 instruments)** | | | | |  |  |  | **Reliability cohort (79 instruments)** | | | | | |
| --- | --- | --- | --- | --- | --- | --- | --- | --- | --- | --- | --- | --- | --- | --- |
| Question | 1 | 2 | 3 | 4 | 5 | <NA> |  | Question | 1 | 2 | 3 | 4 | 5 | <NA> |
| 1 | 3 (0,7%) | 8 (1,8%) | 44 (10%) | 254 (57,6%) | 127 (28,8%) | 5 (1,1%) |  | 1 | 0 (0%) | 1 (0,2%) | 9 (2%) | 48 (10,9%) | 20 (4,5%) | 0 (0%) |
| 2 | 3 (0,7%) | 5 (1,1%) | 36 (8,2%) | 269 (61%) | 120 (27,2%) | 8 (1,8%) |  | 2 | 0 (0%) | 0 (0%) | 11 (2,5%) | 52 (11,8%) | 14 (3,2%) | 1 (0,2%) |
| 3 | 0 (0%) | 9 (2%) | 96 (21,8%) | 247 (56%) | 86 (19,5%) | 3 (0,7%) |  | 3 | 0 (0%) | 1 (0,2%) | 18 (4,1%) | 42 (9,5%) | 16 (3,6%) | 1 (0,2%) |
| 4 | 1 (0,2%) | 8 (1,8%) | 38 (8,6%) | 150 (34%) | 241 (54,6%) | 3 (0,7%) |  | 4 | 0 (0%) | 3 (0,7%) | 4 (0,9%) | 30 (6,8%) | 40 (9,1%) | 1 (0,2%) |
| 5 | 1 (0,2%) | 16 (3,6%) | 121 (27,4%) | 202 (45,8%) | 96 (21,8%) | 5 (1,1%) |  | 5 | 1 (0,2%) | 2 (0,5%) | 17 (3,9%) | 36 (8,2%) | 19 (4,3%) | 3 (0,7%) |
| 6 | 0 (0%) | 10 (2,3%) | 23 (5,2%) | 137 (31,1%) | 264 (59,9%) | 7 (1,6%) |  | 6 | 0 (0%) | 0 (0%) | 4 (0,9%) | 27 (6,1%) | 46 (10,4%) | 1 (0,2%) |
| 7 | 0 (0%) | 5 (1,1%) | 29 (6,6%) | 159 (36,1%) | 237 (53,7%) | 11 (2,5%) |  | 7 | 0 (0%) | 1 (0,2%) | 8 (1,8%) | 23 (5,2%) | 44 (10%) | 2 (0,5%) |
| 8 | 0 (0%) | 2 (0,5%) | 69 (15,6%) | 230 (52,2%) | 126 (28,6%) | 14 (3,2%) |  | 8 | 0 (0%) | 2 (0,5%) | 5 (1,1%) | 43 (9,8%) | 25 (5,7%) | 3 (0,7%) |
| 9 | 1 (0,2%) | 27 (6,1%) | 83 (18,8%) | 132 (29,9%) | 191 (43,3%) | 7 (1,6%) |  | 9 | 1 (0,2%) | 3 (0,7%) | 15 (3,4%) | 20 (4,5%) | 37 (8,4%) | 2 (0,5%) |
| 10 | 1 (0,2%) | 31 (7%) | 105 (23,8%) | 196 (44,4%) | 101 (22,9%) | 7 (1,6%) |  | 10 | 0 (0%) | 6 (1,4%) | 13 (2,9%) | 38 (8,6%) | 20 (4,5%) | 1 (0,2%) |
| 11 | 3 (0,7%) | 17 (3,9%) | 101 (22,9%) | 250 (56,7%) | 60 (13,6%) | 10 (2,3%) |  | 11 | 0 (0%) | 0 (0%) | 18 (4,1%) | 44 (10%) | 13 (2,9%) | 3 (0,7%) |
| 12 | 0 (0%) | 14 (3,2%) | 48 (10,9%) | 230 (52,2%) | 138 (31,3%) | 11 (2,5%) |  | 12 | 0 (0%) | 1 (0,2%) | 8 (1,8%) | 32 (7,3%) | 34 (7,7%) | 3 (0,7%) |
| 13 | 0 (0%) | 2 (0,5%) | 37 (8,4%) | 176 (39,9%) | 212 (48,1%) | 14 (3,2%) |  | 13 | 0 (0%) | 0 (0%) | 6 (1,4%) | 29 (6,6%) | 41 (9,3%) | 2 (0,5%) |
| 14 | 4 (0,9%) | 16 (3,6%) | 19 (4,3%) | 194 (44%) | 198 (44,9%) | 10 (2,3%) |  | 14 | 0 (0%) | 2 (0,5%) | 5 (1,1%) | 35 (7,9%) | 34 (7,7%) | 2 (0,5%) |
| 15 | 15 (3,4%) | 45 (10,2%) | 81 (18,4%) | 166 (37,6%) | 120 (27,2%) | 14 (3,2%) |  | 15 | 4 (0,9%) | 7 (1,6%) | 12 (2,7%) | 28 (6,3%) | 25 (5,7%) | 2 (0,5%) |
