# Supplementary material for: Assessing physicians’ and nurses’ experience of dying and death in the ICU: development of the CAESAR-P and the CAESAR-N instruments
Source: Crit Care. 2020 Aug 25;24:521. doi: 10.1186/s13054-020-03191-z (PMC7448438; doi:10.1186/s13054-020-03191-z)
Supplement: Supplementary file 5 — Additional file 5: Supplemental Table 5. Psychometric validation of the physician questionnaire: Measurement error: internal consistency. [file 13054_2020_3191_MOESM5_ESM.docx]

**Supplemental Table 5**: **Psychometric validation of the physician questionnaire**: **Measurement error: internal consistency**

|  | Learning cohort | Reliability cohort |
| --- | --- | --- |
| Number | 417 | 90 |
| Mean ± sd of inter-item correlation | 0.238±0.058 | 0.251±0.071 |
| Mean ± sd of total-item correlation | 0.537±0.096 | 0.547±0.118 |
| Cronbach a (95%CI bootstrap adjusted) | 0.817 (0.787,0.842) | 0.852 (0.802, 0.892) |
| Split-half reliability adjusted using the Spearman Brown prophecy formula (rBS) | 0.811 (0.770, 0.844) | 0.878 (0.809, 0.922) |
| Composite Reliability using confirmatory factor analysis | 0.828 | 0.837 |

Internal consistency was acceptable with a Cronbach alpha at 0.817 (learning cohort) and 0.852 (reliability cohort)
