# Supplementary material for: Assessing physicians’ and nurses’ experience of dying and death in the ICU: development of the CAESAR-P and the CAESAR-N instruments
Source: Crit Care. 2020 Aug 25;24:521. doi: 10.1186/s13054-020-03191-z (PMC7448438; doi:10.1186/s13054-020-03191-z)
Supplement: Supplementary file 6 — Additional file 6: Supplemental Table 6. Psychometric validation of the nurse questionnaire: Measurement error: internal consistency. [file 13054_2020_3191_MOESM6_ESM.docx]

**Supplemental Table 6: Psychometric validation of the nurse questionnaire**: **Measurement error: internal consistency.**

|  | Learning cohort | Reliability cohort |
| --- | --- | --- |
| Number | 398 | 66 |
| Mean ± sd of inter-item correlation | 0.209±0.051 | 0.223±0.057 |
| Mean ± sd of total-item correlation | 0.511±0.099 | 0.521±0.102 |
| Cronbach a (95%CI bootstrap adjusted) | 0.824 (0.792, 0.853) | 0.823 (0.718, 0.894) |
| Split-half reliability adjusted using the Spearman Brown prophecy formula (rBS) | 0.825 (0.787, 0.857) | 0.822 (0.719, 0.892) |
| Composite Reliability using confirmatory factor analysis | 0.800 | 0.815 |

Internal consistency was acceptable with a Cronbach alpha at 0.824 (learning cohort) and 0.823 (reliability cohort)
